# Novel *WTX* nonsense mutation in a family diagnosed with osteopathia striata with cranial sclerosis: Case report

**DOI:** 10.1097/MD.0000000000027346

**Published:** 2021-10-08

**Authors:** Changhoon Jeong, Myungshin Kim, Jisook Yim, Il-Jung Park, Jiwon Lee, Jaeyoung Lee

**Affiliations:** a Department of Orthopedic Surgery, Bucheon St. Mary's Hospital, College of Medicine, The Catholic University of Korea, Seoul, Republic of Korea; b Department of Laboratory Medicine, College of Medicine, The Catholic University of Korea, Seoul, Republic of Korea.

**Keywords:** bone dysplasia, gene mutation, osteopathia striata with cranical sclerosis

## Abstract

Rationale: Osteopathia striata with cranial sclerosis is characterized by linear striations in the metaphysis of the long bones and pelvis with cranial sclerosis. It is an X-linked dominant sclerosing bone dysplasia and affected males show fetal or neonatal lethality. Mutations in the gene encoding Wilms tumor on the X chromosome (*WTX*) was identified as the cause of X-linked osteopathia striata with cranial sclerosis. About 30 pathogenic mutations in *WTX* have been reported recently. We have identified a novel nonsense mutation in the family diagnosed as osteopathia striata with cranial sclerosis.

**Patient concerns:**

The proband came to our attention at age 9 for the evaluation of toe-out gait and planovalgus deformity. Clinically, the proband showed coarse facial features including frontal bossing, ocular hypertelorism, wide depressed nasal bridge, dental malocclusion, mild macrocephaly and low set ears. Radiologically, sclerotic linear striations were seen in the X-rays of the pelvis and the metaphyseal region of femur and tibia and the cranial sclerosis was observed. The proband's mother presented similar facial features and the X-rays of the pelvis, femur, and tibia revealed same sclerotic linear striations as the proband's.

**Diagnoses:**

Osteopathia striata with cranial sclerosis.

**Interventions:**

A genetic analysis was conducted on genomic DNA isolated from peripheral blood leukocytes of the proband and the mother for confirming the clinical suspicion of osteopathia striata with cranial sclerosis. *WTX* on Xq11.2 gene was analyzed in direct sequencing for coding exons including intron-exon boundaries.

**Outcomes:**

One novel nonsense mutation (c.1003C>T, p.Gln335^∗^) and known single nucleotide variant were observed in a heterozygous form.

**Lessons:**

We found a novel nonsense mutation in a family diagnosed as osteopathia striata with cranial sclerosis. The relationship between various clinical features and genetic mutations can be clarified by accumulation of genetic database.

## 1 Introduction

Osteopathia striata with cranial sclerosis (OSCS) is characterized by linear striations in the metaphysis of long bones and pelvis with cranial sclerosis.^[1]^ It is an X-linked dominant sclerosing bone dysplasia. Affected males show fetal or neonatal lethality.^[2]^ Clinical findings of OSCS include macrocephaly, frontal bossing, wide nasal bridge, hearing difficulty, and abnormal palate.^[3]^ Jenkins et al^[4]^ have reported that mutations in the gene encoding *WTX* (Wilms tumor on the X chromosome; *FAM123B* or *AMER1*), a repressor for WNT signaling, are the cause of X-linked OSCS. About 30 pathogenic mutations in *AMER1* have been reported recently.^[5]^ Here, we report a family with OSCS phenotypes caused by a novel nonsense mutation in *WTX*.

## 2 Case report

The proband came to our attention at age 9 for the evaluation of toe-out gait and planovalgus deformity. Her height was 133 cm (25th percentile). Her bodyweight was 30 kg (31th percentile). Clinically, the proband showed coarse facial features including frontal bossing, ocular hypertelorism, wide depressed nasal bridge, dental malocclusion, mild macrocephaly (head circumference: 63 cm > 2SD), and low set ears. The otologic examination showed severe mixed hearing loss with a wide gap in air conduction on the left side and medium-grade sensory neural hearing loss on the right side.

**Figure 1:**
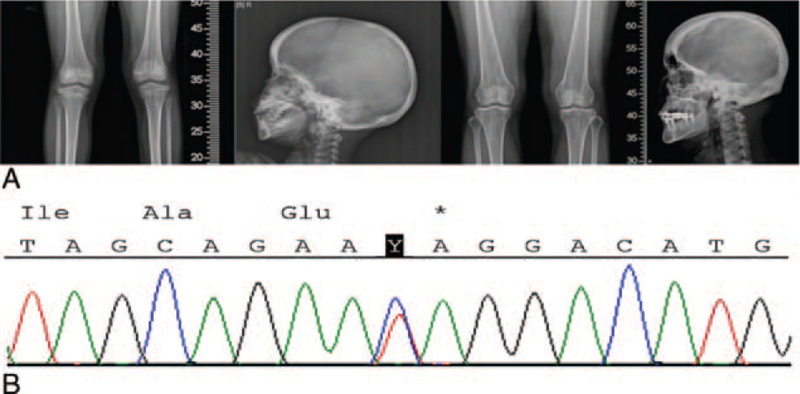
A, Linear striations in long bones and cranial sclerosis of the proband and the mother. B, Electropherogram of a *WTX* mutation. One novel nonsense mutation (c.1003C>T, p.Gln335^∗^) was observed in the proband and the mother. WTX = Wilms tumor on the X chromosome.

Radiologically, sclerotic linear striations were seen in X-rays of the pelvis and the metaphyseal region of femur and tibia. Cranial sclerosis was also observed (Fig. 1A). A pituitary macroadenoma measuring about 8 × 11 × 11 mm was found in magnetic resonance imaging of brain. The pituitary hormonal test was within normal limit.

The proband's mother presented similar facial features. She also had an ocular hypertelorism and dental malocclusion. X-rays of the pelvis, femur, and tibia revealed the same sclerotic linear striations as the proband (Fig. 1A).

Informed consent for gene analysis was obtained from parents of the child. A genetic analysis was conducted using genomic DNAs isolated from peripheral blood leukocytes of the proband and her mother as specimens. Direct sequencing was performed for *WTX* on Xq11.2 to analyze coding exons including intron-exon boundaries. One novel nonsense mutation (c.1003C>T, p.Gln335^∗^) and known single nucleotide variant were observed in a heterozygous form (Fig. 1B). Novel *WTX* nonsense mutation was found based on NM_152424.3 reference sequence. Analysis for the mother of the proband showed the same nonsense mutation and single nucleotide variant.

## 3 Discussion

OSCS is a sclerotic osteodysplasia characterized by thick osteosclerosis in the cortex of long bone metaphysis and pelvis.^[6]^*WTX* mutations located in the long arm of X chromosome are known to be the cause of OSCS. They show X-chromosomal association with dominant genetic patterns.^[4]^ Clinical features of OSCS vary widely within a household. Types accompanied by cranial sclerosis may have features such as hearing impairment, cerebral palsy, cataracts, and delayed development.^[6]^ Genetic variation has been discovered as the cause of OSCS, explaining why OSCS develops variable clinical features not limited to the orthopedic area.

Bony sclerosis is the most prominent radiographic characteristic of OSCS. Recently, several other sclerotic conditions have been explained by increased bone formation due to increased WNT signaling. However, the difference between OSCS and other sclerotic conditions is the striation in the bone. This is believed to be due to differences in bone formation rate. Interestingly, otosclerosis is present in several OSCS patients, indicating increased bone formation in the otic capsule, a bone that in general shows almost complete absence of bone remodeling. It is also known that the WNT/b-catenin signaling pathway plays distinct, even opposing roles during various stages of cardiac development.^[7]^

Typical radiologic and clinical findings of OSCS such as sclerotic linear striations in X-ray and coarse facial features were found in our cases. The Novel *WTX* nonsense mutation could be the new cause of OSCS. Long-term observation is needed to ascertain the expression of other phenotypes of OSCS.

Jenkins et al^[4]^ have reported that germline mutation in *WTX* can cause a sclerosing skeletal dysplasia, although it does not predispose to tumorigenesis. However, Fujita et al^[5]^ have reported a case of OSCS with hepatoblastoma and Sperotto et al^[8]^ have reported case of OSCS with Wilm tumor. Several pathogenic mutations in *WTX* have been reported.^[5]^ Its phenotype–genotype correlation is uncertain. Molecular diagnosis of OSCS in patients with frontal bossing, hearing impairment, and linear striation of long bones in X-ray can be made by identifying responsible mutations of genes.

In conclusion, we found a novel nonsense mutation in a family diagnosed as OSCS. In the future, more genetic variations may be observed for this disease. Relationships between various clinical features and genetic mutations need to be clarified by further studies.

## Author contributions

**Data curation:** Jiwon Lee.

**Investigation:** Jisook Yim.

**Supervision:** Changhoon Jeong, Myungshin Kim.

**Writing – original draft:** Jaeyoung Lee.

**Writing – review & editing:** Il-jung Park.

## References

[1] Ng DW. A case study of a preadolescent with osteopathia striata with cranial sclerosis. J Pediatr Health Care 2017;31:511–6. 28390856 10.1016/j.pedhc.2017.01.003

[2] Vasiljevic A Azzi C Lacalm A. Prenatal diagnosis of osteopathia striata with cranial sclerosis. Prenat Diagn 2015;35:302–4. 25284440 10.1002/pd.4513

[3] Enomoto Y Tsurusaki Y Harada N Aida N Kurosawa K. Novel AMER1 frameshift mutation in a girl with osteopathia striata with cranial sclerosis. Congenit Anom (Kyoto) 2018;58:145–6. 28990699 10.1111/cga.12258

[4] Jenkins ZA van Kogelenberg M Morgan T. Germline mutations in WTX cause a sclerosing skeletal dysplasia but do not predispose to tumorigenesis. Nat Genet 2009;41:95–100. 19079258 10.1038/ng.270

[5] Fujita A Ochi N Fujimaki H. A novel WTX mutation in a female patient with osteopathia striata with cranial sclerosis and hepatoblastoma. Am J Med Genet A 2014;164A:998–1002. 24459086 10.1002/ajmg.a.36369

[6] Perdu B de Freitas F Frints SG. Osteopathia striata with cranial sclerosis owing to WTX gene defect. J Bone Miner Res 2010;25:82–90. 20209645 10.1359/jbmr.090707

[7] Tzahor E. Wnt/beta-catenin signaling and cardiogenesis: timing does matter. Dev Cell 2007;13:10–3. 17609106 10.1016/j.devcel.2007.06.006

[8] Sperotto F Bisogno G Opocher E. Osteopathia striata with cranial sclerosis and Wilms tumor: Coincidence or consequence? Clin Genet 2017;92:674–5. 29120061 10.1111/cge.13082

